# News and Notes

**Published:** 1904-03

**Authors:** 


### *NEWS AND NOTES.*

IT will be of interest to all physicians in the State to learn that a professional nurses' register has been established at Palmer's Viaduct Pharmacy, 11 Peachtree street, Atlanta. Only graduates and competent names are on this register, and one can be obtained by either writing, telegraphing or telephoning them. Phone 64.

---

A NAVAL tuberculosis camp has been established at Pensacola (Fla.) navy yard.

---

THE attention of readers is called to the notice of Special Course of the Chicago Policlinic and Hospital on page xviii.

---

DR. CHAS. WHITNEY DABNEY has accepted the offer of the presidency of the University of Cincinnati. He will take up his new office September 1 next.

---

WAR on the spitting nuisance has been declared by the new Health Commissioner of New York. Officers have been sent to the ferry-houses, where the offense is most flagrant, and a number of the lawbreakers have been arrested.

---

THE American Society of Tropical Medicine held its first formal meeting in the auditorium of Houston Hall at the University of Pennsylvania on the evening of January 9. Dr. James Carroll of the United States army delivered an address entitled "The Etiology of Yellow Fever."

---

MILWAUKEE fears a general invasion of hydrophobia, which has assumed an epidemic form. Dr. Nicholas Senn, of Chicago, has written to the Milwaukee board of health, advising the board to see to it that every dog in Milwaukee running at large shall be promptly killed or muzzled.

---

WE note with interest that the Fourth Annual Summer Course in Medicine of the Tulane University of Louisiana will begin May 2 and last six weeks. Descriptive circular will be mailed on application. Address "Summer Medical Course, Tulane University, P. O. Drawer 261, New Orleans, La."

---

EVEN the indolent Mexicans have recognized the importance of prophylactic health measures for the public good, and the health department has issued several thousand earthenware bowls, containing a mixture of sulphur and saltpeter and suspended by wires in such a way as to admit of their introduction into wells. The authorities hope in this manner to kill off the mosquitoes occupying the wells. The expense of the outfits is borne by the health department of the republic.

---

NOT long ago, says the *New York Medical Journal*, two keepers employed in the Philadelphia Hospital were charged with brutality toward an insane patient. It appeared that rather competent testimony was given by one of the resident physicians, who exerted most laudable efforts toward assisting in the administration of justice. It is now stated that as the only evidence procurable was that of witnesses who themselves were insane, such action would hardly hold in law, and for that reason the case was dropped.

---

THE first issue of a new journal called the *Journal of Infectious Diseases* recently appeared. The new magazine is devoted to the publication of original investigations dealing with the general phenomena, causes, and prevention of infectious diseases. It has been established in connection with the Memorial Institute for Infectious Diseases, founded by Mr. and Mrs. McCormick in memory of their son, who died of scarlet fever. It is estimated that the annual cost for publishing this journal will aggregate $15,000.

---

DR. ARABELLA KENEALY, a practitioner of London, hurls some scathing invectives at the corset as a means of producing a temper as waspish as the waist of the victim. In a recent issue of *The Nineteenth Century and After*, she presents staggering statistics on the evil wrought by the compression-harness, and states her belief that woman will never take her rightful place in the world until she discards the abomination. Perhaps her contention is well founded, but we do not recollect learning that among the people whose women do not compress their waists there is a marked feminine ascendency.—*Exchange.*

---

THE recent trial in an English court of the case of Bayliss *vs.* Coleridge will be noted by all who have the interest of science at heart. Mr. Coleridge publicly charged that Dr. Bayliss tortured animals in his physiology lectures before the students of the London University. He based his accusations upon the statements of two women, Swedish medical students, who attended the lecture at which the tortures were said to have occurred. Dr. Bayliss sued for libel and was awarded $10,000 damages. Such an object-lesson as this may have in the future a tendency to modify the accusations brought by rabid antivivisectionists against the medical profession.

---

AN Indiana physician believes that by means of properly controlled influences on negro infants he can prevent them from turning dark. It is known that negro children are born white, or nearly so, and the reason assigned by the Indiana man for their growing dark later in life is that the skin of the negro is extremely sensitive, throwing out large quantities of pigment as a means of protection. His plan for testing his theory is to have the room in which the experiment is to be carried out draped and furnished in red, with red lights and all attendants dressed in red garments. The infant undergoing the experiment is also to be clad in red. Under these conditions he believes that the child will retain its original white color. The experiment promises to be interesting. We trust that no convalescent from the tremens ward will ever get into the experiment-room by mistake. A relapse would be practically assured.—*Exchange.*

---

The Chicago Bulletin of the Department of Health, in discussing the arbitrary action of the local milk-dealers, says: "The disastrous effects on child life—threatened by the barbarous action of the milk-wagon drivers in refusing to make more than one delivery of milk a day—were largely overcome by the relatively cool weather of the summer, by unusual effort in milk inspection (including the employment of an emergency corps of inspectors known as 'milk scouts'), and by an enormous development of the sterilized milk service fostered by the Woman's Club and Hospital Association." As a matter of record the mortality of infants and young children during 1903 was lower than ever before known—8.8 per cent. less than in 1902, 9.4 per cent. less than in 1901, and 57 per cent. less than the average of the period between the two census years 1890 and 1900. In 1891 the deaths of children under five years of age numbered 110.38 in every 10,000 of population, in 1903 they were only 41.7.

SEVERAL important changes are noted in the brochure of the American Electro-Therapeutic Association, recently issued. The requirements for membership provide that a "candidate for election to ordinary fellowship in the American Electro-Therapeutic Association must be a graduate of a recognized medical college, and a member in good standing of his regular county and state societies." At the meeting last September it was voted that the admission fee should be dropped, and that the five dollars accompanying the candidate's application for membership should constitute the first year's dues, being the only expenditure required of the newly-elected member. The new regulations provide that the Executive Council be empowered to elect candidates to full membership immediately after investigation as to eligibility. By means of this provision candidates may be admitted to the privileges of membership within thirty days after the receipt of their application, instead of being obliged to wait for concerted action at the next ensuing annual meeting. The next (fourteenth) annual convention will be held at St. Louis, Mo., on Tuesday, Wednesday, Thursday and Friday, September 14 to 16, when the scientific meetings will be held in the mornings only, in order to afford members an opportunity of visiting the Exposition in the afternoon, and possibly in the evenings.

---

THE International Congress of Otology will hold its seventh Congress at Bordeaux, August 1 to 4, 1904, under the patronage of the French Minister of Public Instruction. The following subjects are proposed for discussion: (1) The choice of a simple and practical acoumetric formula, to be introduced by Professors Politzer, Gradenigo, and Delseaux. (2) The diagnosis and treatment of suppuration of the labyrinth, to be introduced by Drs. Brieger, Von Stein, and Dundas Grant. (3) Technique of the opening of brain abscess of aural origin and after-treatment, to be introduced by Drs. Kapp, Schmiegelow, and Botey. The Lenval prize given in recognition of the greatest progress achieved in the practical treatment of affections of the auditory apparatus during the interval between two sessions of the Congress, or to the inventor of an easily portable apparatus capable of markedly improving the hearing of deaf people, will be awarded during the Congress. The jury consists of Professor Politzer of Vienna (president), Dr. Benni of Warsaw, Dr. Gelle of Paris, Dr. Urban Pritchard of London, Professor St. John Roosa of New York, Professor Kircher of Wurzburg, Professor Grazi of Florence, and Professor E. J. Moure of Bordeaux. The prize consists of the interest on 3000 francs accumulated during the interval between two sessions of the Congress. Candidates must send in a statement of their claims to Dr. E. J. Moure, president of the Organizing Committee, Cours du Jardin Publique, 25 bis., Bordeaux, before July 1, 1904.

---

### AN UNAPPRECIATED SOURCE OF TYPHOID INFECTION.

P. B. Barringer, Charlottesville, Va. (*N. Y. Medical Record*, December 19, 1903), calls attention to the possibility of infection of the road-bed by the evacuations of patients while on a journey, before they are too sick to be confined to bed. As the number of patients traveling is large, there can easily be seen how the germs may be deposited and kept alive by the moisture of the air and the discharges from trains which continually pass over them. From these foci creeks, springs, etc., can easily be infected. Furthermore, the feces and urine of patients may be discharged directly into a river from which a city takes its water supply. In the railway service, 90 per cent of the typhoid fever patients are track men, i. e., those whose duty it is to keep the rails and ties in good condition, and who therefore are continually exposed to the dust raised by passing trains, which dust might be laden with germs. Furthermore, why might not such a reason lie at the base of the numerous infections of the traveling public?

